# Non-cancer-specific survival in patients with primary central nervous system lymphoma: A multi-center cohort study

**DOI:** 10.3389/fonc.2023.1096027

**Published:** 2023-02-08

**Authors:** Kaiyi Chi, Ruoyun Zhou, Zehao Luo, Hongjun Zhao, Yanting Jiang, Baixin He, Yemin Li, Dongting Chen, Manting Feng, Yinglan Liang, Wenting Yang, Ruisi Liu, Dunchen Yao, Xiaozhen Lin, Xiuhong Xu

**Affiliations:** ^1^ Department of Clinical Medicine, The Second Clinical College of Guangzhou Medical University, Guangzhou, China; ^2^ Cardiovascular Medicine and Cardio-Oncology Group, Medical Exploration and Translation Team, Guangzhou, China; ^3^ Department of Clinical Medicine, The Third Clinical College of Guangzhou Medical University, Guangzhou, China; ^4^ Department of Clinical Medicine, The Sixth Clinical College of Guangzhou Medical University, Guangzhou, China; ^5^ Department of Clinical Medicine, The First Clinical College of Guangzhou Medical University, Guangzhou, China; ^6^ Department of Anesthesiology, The Second Clinical College of Guangzhou Medical University, Guangzhou, China; ^7^ Department of Medical Imageology, The Second Clinical College of Guangzhou Medical University, Guangzhou, China; ^8^ Department of Radiation Oncology, Sun Yat-sen University Cancer Center, Guangzhou, China; ^9^ Department of Cardiology, Guangzhou Institute of Cardiovascular Disease, The Second Affiliated Hospital of Guangzhou Medical University, Guangzhou, China; ^10^ Department of Acupuncture and Massage Rehabilitation, Integrated Hospital of Traditional Chinese Medicine, Southern Medical University, Guangzhou, China

**Keywords:** primary central nervous system lymphoma, primary central nervous system diffuse large B-cell lymphoma, non-cancer-specific survival, risk factors, SEER

## Abstract

**Objective:**

The study aimed to evaluate the non-cancer-specific death risk and identify the risk factors affecting the non-cancer-specific survival (NCSS) in patients with primary central nervous system lymphoma (PCNSL).

**Methods:**

This multi-center cohort study included 2497 patients with PCNSL in the Surveillance, Epidemiology and End Results (SEER) database from 2007 to 2016, with a mean follow-up of 4.54 years. The non-cancer-specific death risk in patients with PCNSL and primary central nervous system diffuse large B-cell lymphoma (PCNS-DLBCL) was evaluated using the proportion of deaths, standardized mortality ratio (SMR), and absolute excess risk (AER). Univariate and multivariate competing risk regression models were utilized to identify the risk factors of NCSS.

**Results:**

PCNSL was the most frequent cause of death in PCNSL patients (75.03%). Non-cancer-specific causes constituted a non-negligible portion of death (20.61%). Compared with the general population, PCNSL patients had higher risks of death from cardiovascular disease (CVD) (SMR, 2.55; AER, 77.29), Alzheimer’s disease (SMR, 2.71; AER, 8.79), respiratory disease (SMR, 2.12; AER, 15.63), and other non-cancer-specific diseases (SMR, 4.12; AER, 83.12). Male sex, Black race, earlier year of diagnosis (2007–2011), being unmarried, and a lack of chemotherapy were risk factors for NCSS in patients with PCNSL and PCNS-DLBCL (all *P* < 0.05).

**Conclusion:**

Non-cancer-specific causes were important competing causes of death in PCNSL patients. More attention is recommended to non-cancer-specific causes of death in the management of PCNSL patients.

## Introduction

Primary central nervous system lymphomas (PCNSL) are highly malignant extranodal lymphomas that originate in and are limited to the central nervous system, including the parenchyma, spinal cord, and meningeal, cranial, and ophthalmic nerves (1). Diffuse large B cell lymphoma (DLBCL), an aggressive histologic subtype, is the most common type in PCNSL (90%) (2). The treatment of PCNSL has dramatically evolved over recent years (3). However, patients with PCNSL still have a poor prognosis, with a 5-year overall survival (OS) rate of 28.6%, which is lower than that of patients with lymphomas originating at other locations (4). The rates of non-cancer-specific causes of death in cancer patients are high, suggesting that determining the risk factors for non-cancer-specific death can improve patient outcomes (5). Likewise, the risk of non-cancer-specific death in PCNSL patients should not be negligible and deserves more attention.

To date, most studies of prognostic risk factors in patients with PCNSL have focused on OS and cancer-specific survival (CSS) (6–8) and ignored non-cancer-specific survival (NCSS). In addition, the analysis of death causes of patients with primary central nervous system diffuse large B-cell lymphoma (PCNS-DLBCL) has not been reported. A previous study evaluated the risk of cardiovascular disease (CVD) death in PCNSL patients with cancer therapy but did not systematically analyze NCSS in PCNSL patients (9). Therefore, further studies are needed to comprehensively analyze the risk of non-cancer-specific death and identify risk factors of NCSS in patients with PCNSL.

Because PCNSL is rare, it is difficult to conduct a large randomized controlled trial (10). National Cancer Institute’s Surveillance, Epidemiology, and End Results (SEER) program contains patient data from 18 registries in the United States (11), which can fulfill the deficiency of limited cases reported in a single center. We conducted a multi-center retrospective study based on SEER to comprehensively evaluate the risk of non-cancer-specific death in patients with PCNSL and identify risk factors of NCSS. These findings may provide better insights and a scientific basis for improving the prognosis and management of patients with PCNSL.

## Methods

### Study participants

Data were obtained from the SEER database, an authoritative data system that covers about 30% of the population of the United States (12). Institutional review board approval was not required for publicly available information (13, 14). Patients in the SEER database initially diagnosed with PCNSL from 2007 to 2016 were included. Patients were selected if they were diagnosed with a single, primary non-Hodgkin’s lymphoma of the central nervous system from 2007–2016 and subsequently followed up. Patients were excluded if their cause of death, race, or marital status was unknown.

### Participant variables and outcomes

Participant variables included sex (male/female), race (White/Black/others), age at diagnosis (< 60 years/≥ 60 years) (15), year of diagnosis (2007–2011/2012–2016), marital status (married/unmarried), pathological type (DLBCL/others), surgery (yes/no evidence), radiotherapy (yes/no evidence), and chemotherapy (yes/no evidence) (9). Other races included American Indian/Alaska Native and Asian/Pacific Islander. Other pathological types included precursor non-Hodgkin B-cell lymphoma; chronic/small lymphocytic leukemia/lymphoma; mantle-cell lymphoma; lymphoplasmacytic lymphoma; intravascular large B-cell lymphoma; Burkitt lymphoma/leukemia; extranodal margin zone lymphoma (MZL); mucosa-associated lymphoid tissue (MALT) cell lymphoma; follicular lymphoma; plasmacytoma; multiple myeloma/plasma-cell leukemia; non-Hodgkin 1ymphoma, B-cell, not otherwise specified (NOS); peripheral T-cell lymphoma, NOS; anaplastic large cell lymphoma, T-/null-cell lymphoma; adult T-cell leukemia/lymphoma; and non-Hodgkin lymphoma, NOS, unknown lineage (12).

NCSS was defined as the period from the date of diagnosis to death from non-cancer-specific causes (5). Follow-up time was calculated as the period from the date of diagnosis with PCNSL until the date of death or last follow-up on December 31, 2016.

### Statistical analysis

Categorical variables were compared using the χ2 test. Standardized mortality ratios (SMRs) and absolute excess risks (AERs) were estimated for non-cancer-specific causes of death after PCNSL diagnosis and compared with the general population. SMRs refer to the ratio of observed to expected deaths (16, 17). AERs were calculated as AERs = 10,000 ([number observed–number expected]/[person−years at risk]) (18), reflecting the absolute increase in the risk of non-cancer-specific causes in the population. Univariate and multivariate competing risk models were used to analyze the risk factors of NCSS. For fully adjusted for baseline, the inclusion criteria that factors were included in multivariate competing risk models were statistically significant factors according to univariate analysis or factors with potential prognostic effect (19–21). All statistical analyses were performed using SEER*Stat (version 8.4.0), SPSS (version 25.0), and Stata (version 15.0) software, with a *P* of < 0.05 defined as statistically significant.

## Results

### Baseline information

A review of the SEER database identified a total of 2497 eligible patients diagnosed with PCNSL from 2007–2016. Evaluation of their demographic characteristics showed that 60.9% of patients were aged ≥ 60 years, and 39.1% of patients were aged < 60 years. The proportion of male patients (53.2%) was higher than that of female patients (46.8%). Of all patients, 78.3% of patients were White, 8.6% of patients were Black, and 13.1% of patients were of other races. A total of 58.2% of patients were unmarried. Additionally, 51.3% of patients were diagnosed in 2007–2011, and 48.7% of patients in 2012–2016. Evaluation of their clinical characteristics showed that 82.8% of these patients had DLBCL, 42.1% of patients underwent surgery, 28.8% of patients received radiotherapy, and 71.6% of patients received chemotherapy (**Table 1**). The mean follow-up time was 4.54 ± 0.08 years.

**Table 1:** Baseline characteristics.

Variable	Patients (N=2497)	
	Number	Proportion
Sex		
Male	1329	53.2%
Female	1168	46.8%
Race		
White	1955	78.3%
Black	215	8.6%
Others^#^	327	13.1%
Age at diagnosis, year		
<60	977	39.1%
≥60	1520	60.9%
Year of diagnosis		
2007-2011	1281	51.3%
2012-2016	1216	48.7%
Marital status		
Married	1044	41.8%
Unmarried	1453	58.2%
Pathological type		
Diffuse Large B-cell Lymphoma	2068	82.8%
Others	429	17.2%
Surgery		
Yes	1051	42.1%
No Evidence	1446	57.9%
Radiotherapy		
Yes	719	28.8%
No Evidence	1778	71.2%
Chemotherapy		
Yes	1787	71.6%
No Evidence	710	28.4%

^#^Others include American Indian/Alaska Native and Asian/Pacific Islander.

### Distribution of cause of death

**Figure 1:**
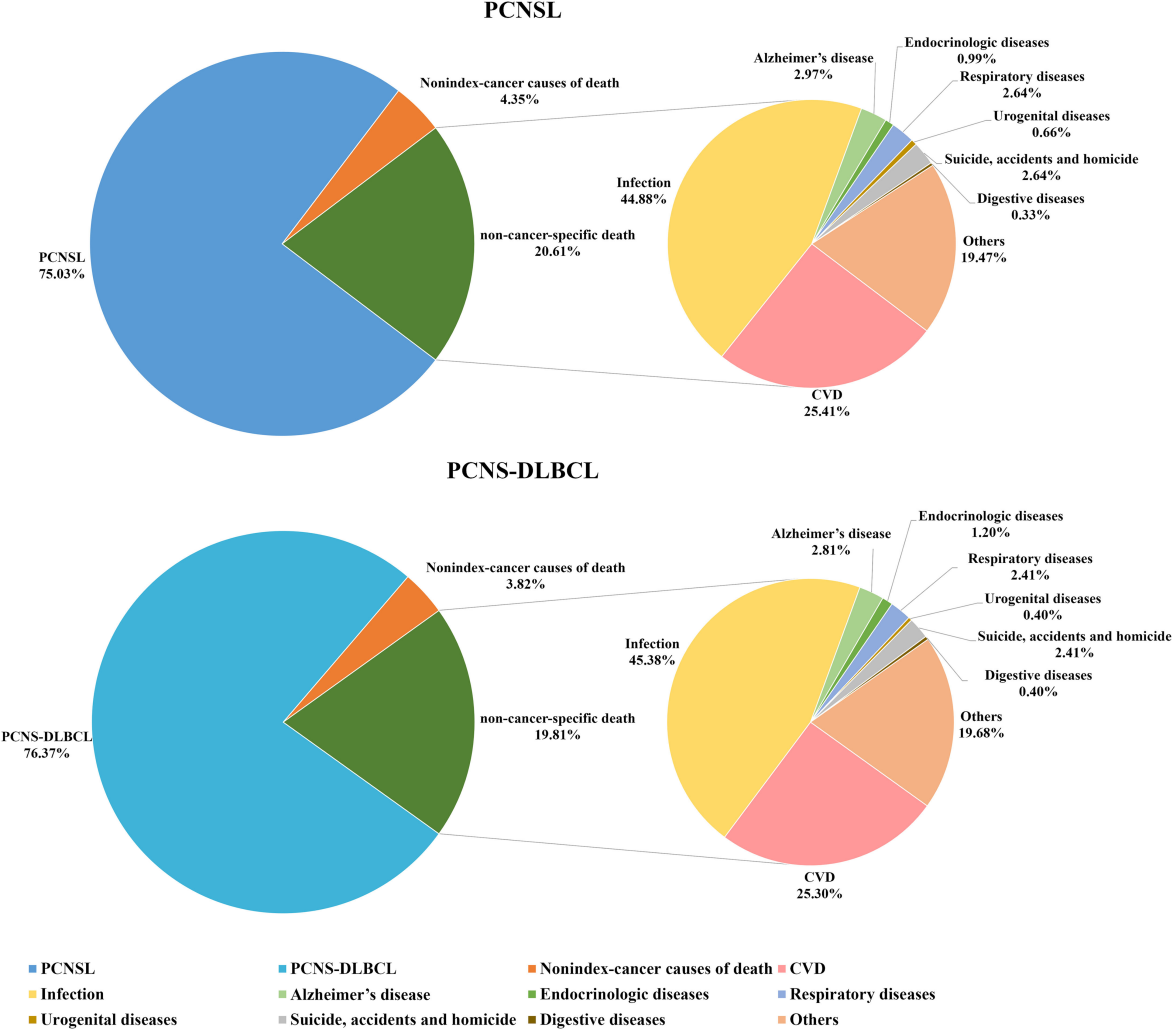
Proportions of deaths in patients with PCNSL and PCNS-DLBCL. PCNSL, primary central nervous system lymphoma; PCNS-DLBCL, primary central nervous system diffuse large B-cell lymphoma.

The leading cause of death in PCNSL patients was primary cancer, observed in 75.03% of patients who died. Non-cancer-specific causes of death were less common, occurring in 20.61% of patients, with 4.35% of patients dying from other cancers. Evaluation of the non-cancer-specific causes of death showed that 44.88% of patients died of infectious diseases, 25.41% of patients died of CVD, and 2.97% of patients died of Alzheimer’s disease. In patients with PCNS-DLBCL, we found that non-cancer-specific causes of death accounted for 19.81% of the overall death rate (**Figure 1**).

### Risks of non-cancer-specific causes of death

Compared with the general population, patients with PCNSL had higher risks of death from CVD (SMR, 2.55; 95% CI, 2.00–3.21; AER, 77.29), Alzheimer’s disease (SMR, 2.71; 95% CI, 1.17–5.34; AER, 8.79), respiratory disease (SMR, 2.12; 95% CI, 1.23–3.39; AER, 15.63), and other non-cancer-specific diseases (SMR, 4.12; 95% CI, 3.17–5.27; AER, 83.12). Evaluation of the risks of short-term and long-term mortality from CVD showed that these risks were especially elevated for diseases of the heart (SMR, 2.24; 95% CI, 1.65–2.95; AER, 47.17), hypertension without heart disease (SMR, 5.03; 95% CI, 1.63–11.73; AER, 6.98), and cerebrovascular diseases (SMR, 4.00; 95% CI, 2.41–6.24; AER, 24.82) (**Table 2**). Compared with the general population, patients with PCNS-DLBCL had higher risks of death from CVD (SMR, 2.24; 95% CI, 1.67–2.93; AER, 65.15), Alzheimer’s disease (SMR, 2.88; 95% CI, 1.16–5.94; AER, 10.37), and other non-cancer-specific diseases (SMR, 3.22; 95% CI, 2.30–4.39; AER, 62.55) (**Table 3**).

**Table 2:** SMRs and AERs for non-cancer-specific causes of death after PCNSL diagnosis.

Causes of death	Timing of deaths after diagnosis							
	2-11 months		12-59 months		60+ months		Total	
	SMR (95% Cl)	AER	SMR (95%Cl)	AER	SMR (95%Cl)	AER	SMR (95%Cl)	AER
CVD	3.99^#^ (2.73-5.63)	157.74	1.82^#^ (1.22-2.62)	39.21	2.56^#^ (1.32-4.46)	82.83	2.55^#^ (2.00-3.21)	77.29
Diseases of the heart	3.58^#^ (2.25-5.43)	104.36	1.56 (0.94-2.43)	20.31	2.24 (0.97-4.42)	50.29	2.24^#^ (1.65-2.95)	47.17
Hypertension without heart disease	11.08^#^ (2.29-32.39)	17.96	1.83 (0.05-10.17)	1.35	5.67 (0.14-31.59)	9.34	5.03^#^ (1.63-11.73)	6.98
Cerebrovascular diseases	5.23^#^ (2.10-10.78)	37.25	3.45^#^ (1.58-6.54)	19.13	3.73 (0.77-10.91)	24.90	4.00^#^ (2.41-6.24)	24.82
Infection	5.19 (0.63-18.76)	10.63	2.56 (0.31-9.26)	3.65	–	-2.60	2.87 (0.78-7.34)	4.54
Diabetes mellitus	3.68 (0.76-10.75)	14.37	1.21 (0.15-4.38)	1.04	–	-5.34	1.70 (0.55-3.97)	3.59
Alzheimer’s (ICD-9 and 10 only)	3.76 (0.78-10.99)	14.49	1.27 (0.15-4.58)	1.26	5.20^#^ (1.07-15.19)	27.47	2.71^#^ (1.17-5.34)	8.79
Respiratory diseases	4.04^#^ (1.85-7.67)	44.55	1.34 (0.49-2.91)	4.54	1.52 (0.18-5.49)	7.75	2.12^#^ (1.23-3.39)	15.63
Digestive diseases	2.56 (0.06-14.26)	4.01	2.36 (0.29-8.52)	3.45	–	-2.60	2.04 (0.42-5.97)	2.67
Nephritis, nephrotic syndrome and nephrosis	4.17 (0.50-15.06)	10.00	–	-2.86	–	-3.21	1.16 (0.14-4.21)	0.49
Suicide, accidents and homicides	4.12^#^ (1.34-9.60)	24.91	1.11 (0.23-3.25)	0.91	1.28 (0.03-7.11)	2.45	1.92 (0.88-3.64)	7.50
Other non-cancer-specific diseases	8.22^#^ (5.69-11.48)	196.48	2.69^#^ (1.71-4.04)	43.31	2.30 (0.84-5.01)	38.47	4.12^#^ (3.17-5.27)	83.12

SMR, Standardized mortality ratio; AER, absolute excess risk; PCNSL, primary central nervous system lymphomas; CI, Confidence interval.

^#^P<0.05.

**Table 3:** SMRs and AERs for non-cancer-specific causes of death after PCNS-DLBCL diagnosis.

Causes of death	Timing of deaths after diagnosis									
	2-11 months		12-59 months			60+ months			Total	
	SMR (95%Cl)	AER	SMR (95%Cl)		AER	SMR (95%Cl)		AER	SMR (95%Cl)	AER
CVD	3.30^#^ (2.04-5.05)	122.30	1.61 (0.99-2.45)		30.85	2.61^#^ (1.25-4.81)		95.53	2.24^#^ (1.67-2.93)	65.15
Diseases of heart	3.50^#^ (2.04-5.60)	101.39	1.40 (0.76-2.34)		15.50	2.43 (0.98-5.00)		63.69	2.14^#^ (1.51-2.94)	45.87
Hypertension without heart disease	4.67 (0.12-26.02)	6.56	2.20 (0.06-12.27)		2.13	6.67 (0.17-37.15)		13.15	3.67 (0.76-10.72)	4.95
Cerebrovascular diseases	2.82 (0.58-8.25)	16.18	2.77^#^ (1.02-6.03)		14.93	2.98 (0.36-10.76)		20.56	2.82^#^ (1.41-5.05)	16.09
Infection	6.45 (0.78-23.29)	14.11	3.12 (0.38-11.27)		5.29	–		-2.87	3.52 (0.96-9.01)	6.49
Diabetes mellitus	3.02 (0.37-10.9)	11.17	1.47 (0.18-5.31)		2.49	–		-5.91	1.66 (0.45-4.26)	3.61
Alzheimer’s disease	4.92^#^ (1.01-14.38)	19.97	1.52 (0.18-5.48)		2.66	4.01 (0.49-14.47)		23.22	2.88^#^ (1.16-5.94)	10.37
Respiratory diseases	3.92^#^ (8.07-43.54)	43.54	0.82 (0.17-2.38)		-2.64	0.96 (0.02-5.35)		-0.64	1.69 (0.84-3.02)	10.18
Digestive diseases	3.15 (0.08-17.54)	5.70	2.95 (0.36-10.65)		5.15	–		-2.67	2.57 (0.53-7.50)	4.15
Nephritis, nephrotic syndrome and nephrosis	2.61 (0.07-14.53)	5.15	–		-3.07	–		-3.61	0.71 (0.02-3.96)	-0.92
Suicide, accidents and homicides	4.21^#^ (1.15-10.77)	25.47	0.48 (0.01-2.66)		-4.26	–		-9.00	1.38 (0.45-3.22)	3.11
Other non-cancer-specific diseases	6.72^#^ (4.21-10.18)	156.43	2.00^#^ (1.09-3.35)		27.21	1.89 (0.51-4.83)		29.07	3.22^#^ (2.30-4.39)	62.55

SMR, Standardized mortality ratio; AER, absolute excess risk; PCNS-DLBCL, primary central nervous system diffuse large B-cell lymphoma; CI, Confidence interval.

^#^P<0.05.

### Risk factors for non-cancer-specific survival

Univariate competitive risk model analyses showed that male sex, Black race, age at diagnosis < 60 years, earlier year of diagnosis (2007–2011), being unmarried, and lack of chemotherapy were related to worse NCSS in patients with PCNSL (**Supplementary Figure S1**, **Supplementary Figure S2** and **Table 4**). Multivariate competitive risk model analyses further showed that male sex (hazard ratio [HR] = 1.45, 95% confidence interval [CI] = 1.11–1.89), Black race (HR = 2.06, 95% CI = 1.43–2.96), earlier year of diagnosis (2007–2011) (HR = 1.59, 95% CI = 1.20–2.10), being unmarried (HR = 1.70, 95% CI = 1.31–2.20), and lack of chemotherapy (HR = 2.11, 95% CI = 1.60–2.78) were risk factors for NCSS in patients with PCNSL (**Table 4**). Univariate competitive risk model analyses showed that male sex, Black race, age at diagnosis < 60 years, earlier year of diagnosis (2007–2011), being unmarried, and lack of chemotherapy were related to worse NCSS in patients with PCNS-DLBCL (**Supplementary Figure S3**, **Supplementary Figure S4** and **Table 5**). Multivariate competitive risk model analyses showed that male sex (HR = 1.49, 95% CI = 1.12–2.00), Black race (HR = 2.19, 95% CI = 1.46–3.29), earlier year of diagnosis (2007–2011) (HR = 1.51, 95% CI = 1.10–2.06), being unmarried (HR = 1.77, 95% CI = 1.33–2.33), and lack of chemotherapy (HR = 2.29, 95% CI = 1.69–3.08) were risk factors for NCSS in patients with PCNS-DLBCL (**Table 5**).

**Table 4:** Competing risk regression analyses of risk factors for non-cancer-specific survival in PCNSL.

Variable	Univariate analysis		Multivariate analysis	
	HR (95% CI)	*P* value	HR (95% CI)	*P* value
Sex				
Male	1.33 (1.03-1.72)	**0.027**	1.45 (1.11-1.89)	**0.006**
Female	Reference		Reference	
Race				
White	Reference		Reference	
Black	2.52 (1.78-3.58)	**0.001**	2.06 (1.43-2.96)	**<0.001**
Others^#^	0.70 (0.45-1.10)	0.122	0.74 (0.47-1.15)	0.182
Age at diagnosis, year				
<60	Reference		Reference	
≥60	0.77 (0.60-0.99)	**0.043**	0.94 (0.72-1.22)	0.716
Year of diagnosis				
2007-2011	1.65 (1.26-2.16)	**<0.001**	1.59 (1.20-2.10)	**0.001**
2012-2016	Reference		Reference	
Marital status				
Married	Reference		Reference	
Unmarried	1.94 (1.50-2.51)	**<0.001**	1.70 (1.31-2.20)	**<0.001**
Pathological type				
Diffuse Large B-cell Lymphoma	0.98 (0.70-1.37)	0.910	1.20 (0.84-1.71)	0.312
Others	Reference		Reference	
Surgery				
Yes	Reference		Reference	
No Evidence	1.23 (0.95-1.60)	0.116	1.24 (0.95-1.61)	0.115
Radiotherapy				
Yes	Reference		Reference	
No Evidence	0.79 (0.61-1.03)	0.087	1.02 (0.77-1.35)	0.870
Chemotherapy				
Yes	Reference		Reference	
No Evidence	2.22 (1.71-2.88)	**<0.001**	2.11 (1.60-2.78)	**<0.001**

PCNSL, primary central nervous system lymphomas; HR, Hazard Ratio; CI, Confidence interval.

Others include American Indian/Alaska Native and Asian/Pacific Islander.

Bold values indicate the results are statistically significant.

**Table 5:** Competing risk regression analyses of risk factors for non-cancer-specific survival in PCNS-DLBCL.

Variable	Univariate analysis		Multivariate analysis	
	HR (95% CI)	*P* value	HR (95% CI)	*P* value
Sex				
Male	1.41 (1.06-1.89)	**0.018**	1.49 (1.12-2.00)	**0.007**
Female	Reference		Reference	
Race				
White	Reference		Reference	
Black	2.98 (2.00-4.41)	**<0.001**	2.19 (1.46-3.29)	**<0.001**
Others#	0.79 (0.45-1.26)	0.330	0.84 (0.53-1.34)	0.465
Age at diagnosis, year				
<60	Reference		Reference	
≥60	0.71 (0.53-0.94)	**0.015**	0.87 (0.65-1.16)	0.351
Year of diagnosis				
2007-2011	1.61 (1.19-2.17)	**0.002**	1.51 (1.10-2.06)	**0.010**
2012-2016	Reference		Reference	
Marital status				
Married	Reference		Reference	
Unmarried	2.07 (1.56-2.74)	**<0.001**	1.77 (1.33-2.33)	**<0.001**
Surgery				
Yes	Reference		Reference	
No Evidence	1.15 (0.86-1.53)	0.340	1.11 (0.83-1.47)	0.481
Radiotherapy				
Yes	Reference		Reference	
No Evidence	0.75 (0.56-1.00)	0.050	0.97 (0.72-1.31)	0.845
Chemotherapy				
Yes	Reference		Reference	
No Evidence	2.51 (1.89-3.36)	**<0.001**	2.29 (1.69-3.08)	**<0.001**

PCNS-DLBCL, primary central nervous system diffuse large B-cell lymphoma; HR, Hazard Ratio; CI, Confidence interval.

Others include American Indian/Alaska Native and Asian/Pacific Islander.

Bold values indicate the results are statistically significant.

## Discussion

This multi-center retrospective cohort study showed that non-cancer-specific causes were an important competing cause of death in PCNSL patients. This study found that 20.61% of patients with PCNSL died of non-cancer-related causes, such as infection, CVD, and Alzheimer’s disease. Similarly, a large population-based study has reported that the risks of non-cancer-related deaths were significantly higher than that of cancer-related deaths after most cancer diagnoses (5). However, that study did not describe conditions related to PCNSL. Our findings were consistent with that earlier report, suggesting that deaths in these patients might be driven by chronic comorbid conditions and acute or iatrogenic infections (5). These results showed that more efforts should be made to manage both cancer- and non-cancer-specific risks of death that contribute to prognosis in these patients.

As important social demographic variables, sex and race were usually considered in previous studies to personalize the treatment of cancer. In line with our results, a recent study showed that sex and race are prognostic factors for OS (22). However, it did not mention the effect on NCSS. In this study, we demonstrated that male and Black race were risk factors for NCSS in PCNSL and PCNS-DLBCL patients. This may be due to gender-biased differences in the X chromosome, hormone levels, and metabolic pathways (23, 24). For race, worse NCSS in those of Black race has been possibly attributed to a higher rate of human immunodeficiency virus (HIV) infection (25), lower economic income, poorer residential environment, and a lack of insurance (26). These factors may result in reduced access to medical resources for diagnosis, treatment, and follow-up, leading to non-cancer-specific death. Understanding the impact of sex and race on NCSS may provide useful information for patients to predict disease progression. Hence, oncologists and neurologists should improve awareness and pay attention to the prevention of non-cancer-specific death of males and those of Black race with PCNSL and further perfect the management strategy.

Marital status is one of the important psychosocial factors but has been ignored in PCNSL (20, 27). In the present study, unmarried PCNSL and PCNS-DLBCL patients had reduced NCSS than married patients, suggesting that marriage plays a beneficial role through social and emotional support (20, 27, 28). Married patients have greater psychological and financial support from their spouses, enabling patients to better relieve mental distress and share finances (29). Additionally, patient adherence to treatment plans and lifestyle habits may depend on the supervision of a partner or spouse during treatment, thus, improving their prognosis (20).

The year of diagnosis is related to NCSS in PCNSL and PCNS-DLBCL patients, with the last 5 years (2012–2016) having a greater NCSS than the first 5 years (2007–2011) of diagnosis. This improvement in NCSS might be attributed to improved PCNSL regimens, resulting in reduced toxicity and, therefore, improved patient prognosis (30). Indeed, 2012 was a time point related to the recommendations of whole brain radiotherapy (WBRT), which was considered a preferred treatment for PCNSL patients with a poor general condition and those who do not respond after systemic high-dose methotrexate (HD-MTX) or relapse in a short time (31). Additionally, first-line treatment with HD-MTX and autologous stem cell transplantation has been shown to be feasible and effective since 2011 (2). Supporting evidence has also been derived from a multi-center retrospective study (9), which concluded that the risks of CVD-associated deaths were 50% lower in PCNSL patients diagnosed in 2010–2015 than in those diagnosed in 2004–2009.

The present study found that chemotherapy could improve NCSS in PCNSL and PCNS-DLBCL patients. This is consistent with a previous study, showing that chemotherapy was related to lower cardiovascular death risk of PCNSL patients (9). HD-MTX is the first-line chemotherapy treatment for PCNSL patients, which also causes neurotoxicity (32). Decreasing toxicity and side effects of chemotherapy regimens for PCNSL over time may contribute to improved NCSS (30). Most physicians use WBRT as part of their usual care for patients with PCNSL to control the high risk of neurotoxicity associated with chemotherapy (33). Rituximab, a monoclonal antibody targeting the B-cell surface antigen CD20, has significantly improved the efficacy and clinical outcome of DLBCL and has been included in the first-line treatment regimen for PCNSL (33, 34). Moreover, high-dose chemotherapy with stem-cell rescue (HDC-ASCT), as a promising consolidative strategy, is also introduced and used among patients with sufficient organ function (35). Our study stressed the important role of chemotherapy in patients with PCNSL and provided a scientific basis for further treatment.

HIV status may have confounding effects on our results, but it is still uncertain and need to be further studied. It should be noted that the pooled prevalence of HIV infection among PCNSL patients was only 6.1% (36), suggesting that the effects of HIV status on our results may be limited. The association among HIV infection and younger age (37, 38), being unmarried (39, 40) as well as chemotherapy (6, 41, 42) is still disputable.

Although DLBCL is an aggressive subtype, the proportion of non-cancer-specific deaths (19.81%) is not low. We, for the first time, studied the prognostic factors of NCSS in PCNS-DLBCL. These findings highlight the importance of non-cancer-specific death risk in PCNS-DLBCL patients. More attention should be paid to minimizing the risk of non-cancer-specific death causes during and after lymphoma treatment and throughout cancer survivorship (36).

## Strengths and limitations

The strengths of our study were its multi-center design and long-term follow-up. To our knowledge, this study is the largest to evaluate the risk of NCSS in patients with PCNSL. Long-term follow-up allowed assessments of the short- and long-term risks of non-cancer-specific death. However, the present study had several limitations. First, the SEER database lacks some of the clinical information related to PCNSL prognoses, such as the results of the cerebrospinal fluid examination, serum lactate dehydrogenase levels, International Extranodal Lymphoma Study Group score and Memorial Sloan Kettering Cancer Center score, and Karnofsky performance scores, which might have resulted in statistical bias. Furthermore, the SEER database lacks detailed information on treatment, such as stem cell transplant, the types and doses of chemotherapy drugs, radiotherapy doses, and duration of treatment. The lack of information on HIV status in SEER prevents further study of its impact on the NCSS in PCNSL patients. Despite these limitations, the present study included a large amount of real and reliable data, suggesting that the findings have clinical reference value.

## Conclusion

Non-cancer-specific causes were important competing causes of death in PCNSL patients. Male sex, Black race, earlier year of diagnosis (2007–2011), being unmarried, and a lack of chemotherapy were risk factors for NCSS in patients with PCNSL and PCNS-DLBCL. Efforts should be made to manage non-cancer-specific death risks that contribute to prognosis in these patients.

## Data availability statement

Publicly available datasets were analyzed in this study. This data can be found here: http://seer.cancer.gov.

## Ethics statement

Institutional review board approval was not required for publicly available information.

## Author contributions

KC: study design, data collection, analysis, interpretation of results, figure design, drafting of the manuscript, and review and editing of the manuscript. RZ, ZL, and HZ: analysis, interpretation of results, drafting of the manuscript, and review and editing of the manuscript. YJ, BH, YML, and DC: interpretation of results and drafting of the manuscript writing. MF, YLL, WY, and RL: study design and drafting of the manuscript; DY: data collection and editing of the manuscript. XL and XX: conception, funding acquisition, project administration and supervision, and review and editing of the manuscript. All authors contributed to the article and approved the submitted version.
