# Supplementary material for: Non-cancer-specific survival in patients with primary central nervous system lymphoma: A multi-center cohort study
Source: Front Oncol. 2023 Feb 8;13:1096027. doi: 10.3389/fonc.2023.1096027 (PMC9945279; doi:10.3389/fonc.2023.1096027)
Supplement: Supplementary file 1 [file DataSheet_1.docx]

**Supplementary figure captions**

**Figure S1.** Cumulative non-cancer-specific mortality in patients with primary central nervous system lymphoma

**Figure S2.** Cumulative non-cancer-specific mortality in patients with primary central nervous system lymphoma (negative results)

**Figure S3.** Cumulative non-cancer-specific mortality in patients with primary central nervous system diffuse large B-cell lymphoma

**Figure S4.** Cumulative non-cancer-specific mortality in patients with primary central nervous system diffuse large B-cell lymphoma (negative results)

**
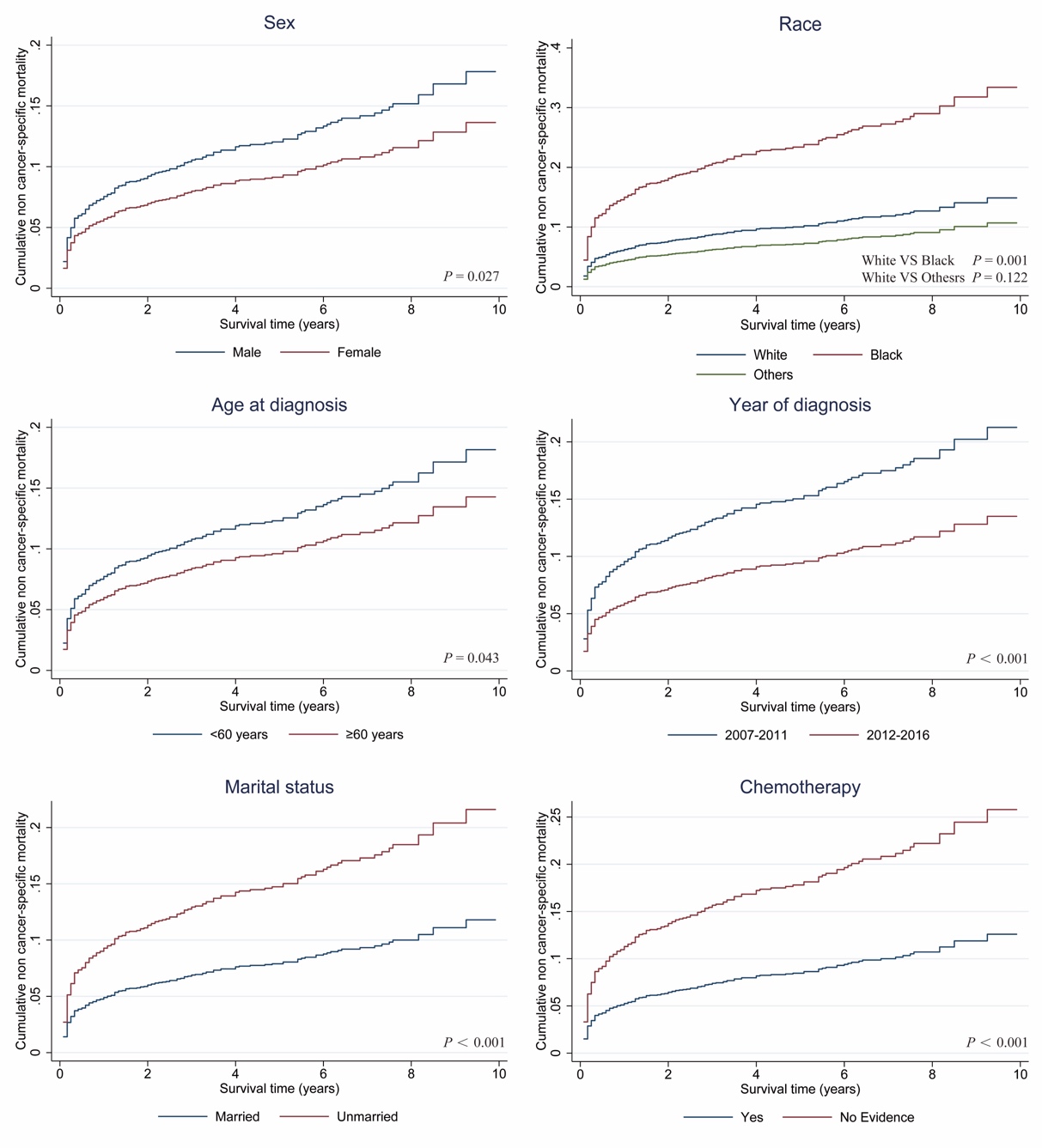
**

**Figure S1. Cumulative non-cancer-specific mortality in patients with primary central nervous system lymphoma**

Sex and age at diagnosis curves are very similar, but have different HRs and cumulative risks.


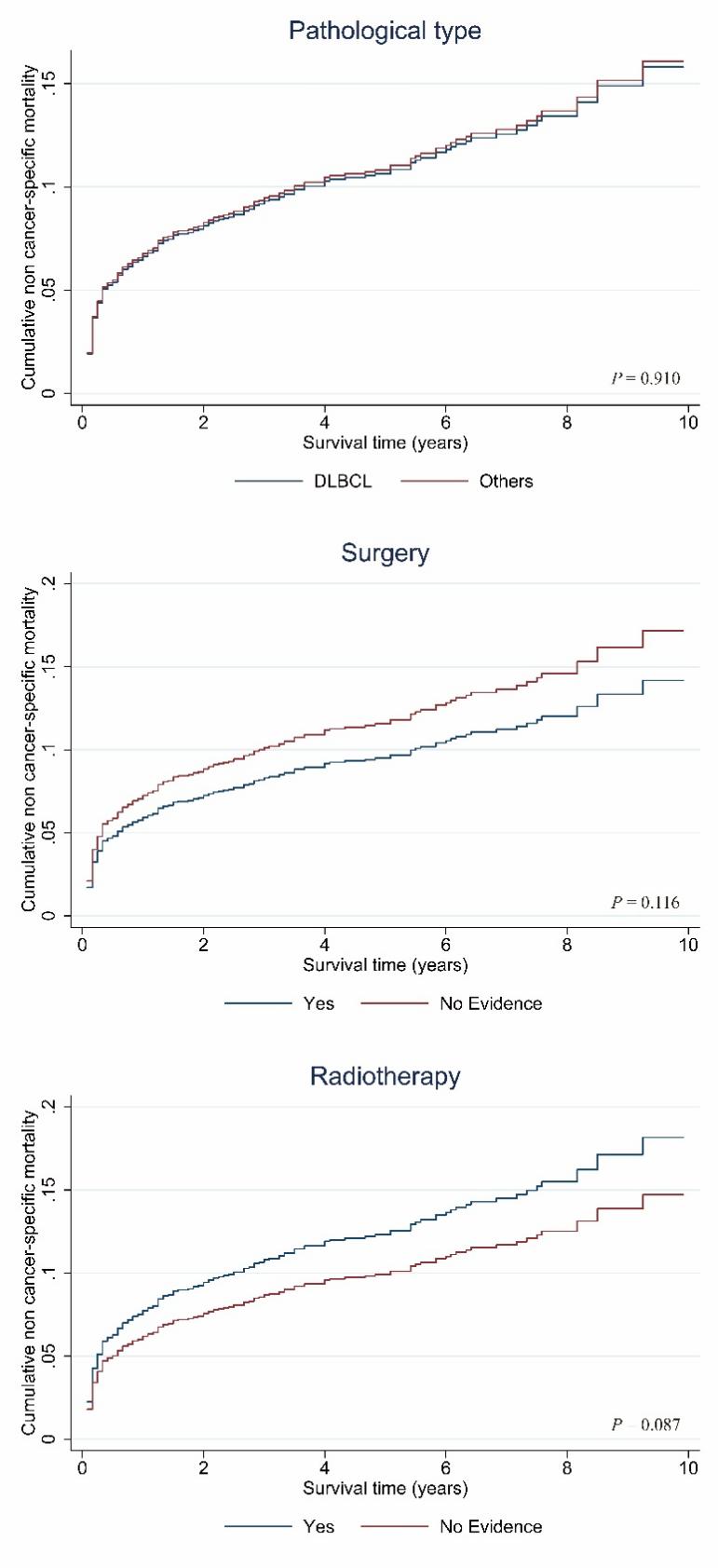


**Figure S2. Cumulative non-cancer-specific mortality in patients with primary central nervous system lymphoma (negative results)**


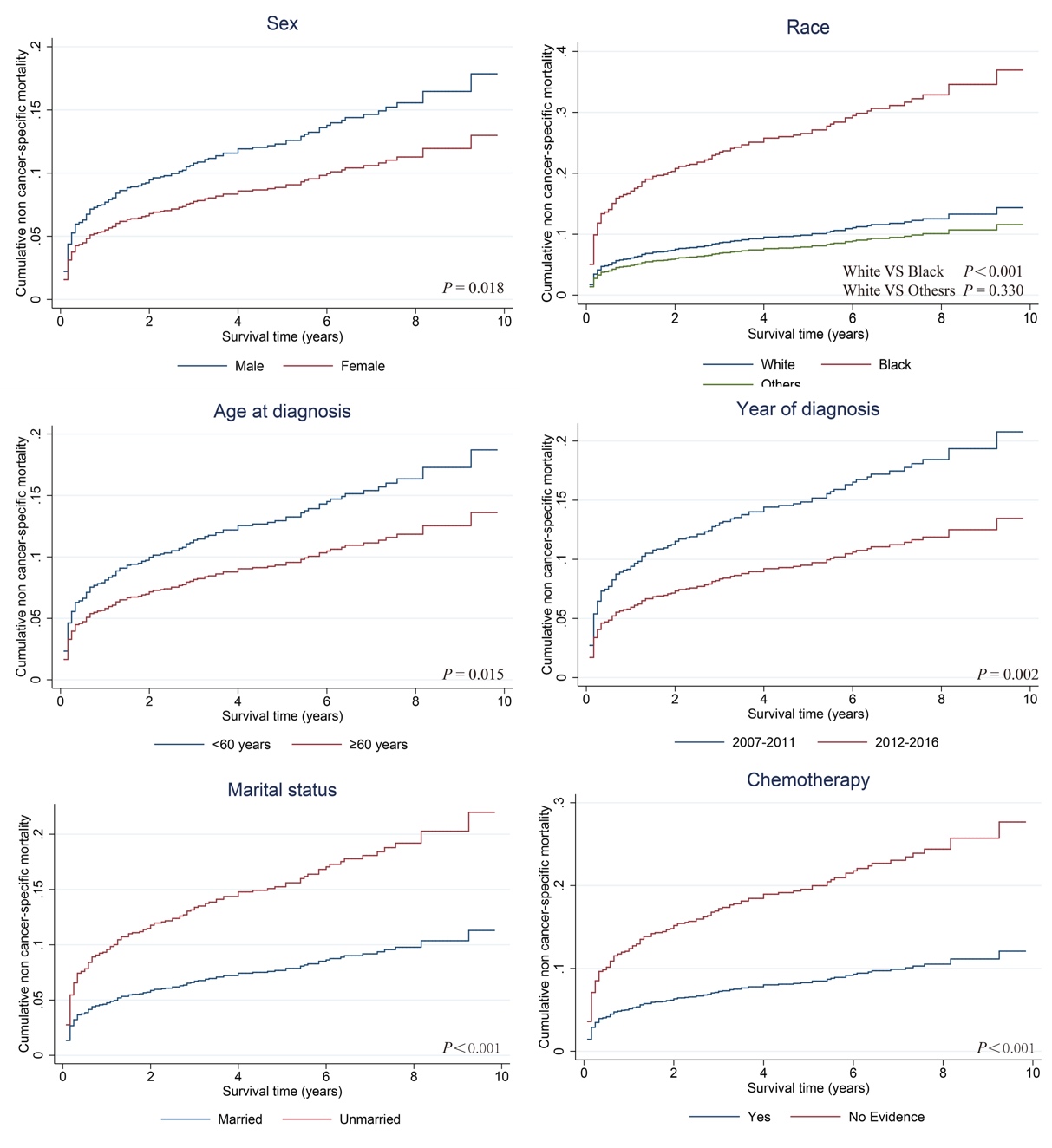


**Figure S2. Cumulative non-cancer-specific mortality in patients with primary central nervous system diffuse large B-cell lymphoma**

Sex and age at diagnosis curves are similar, but have different HRs and cumulative risks.


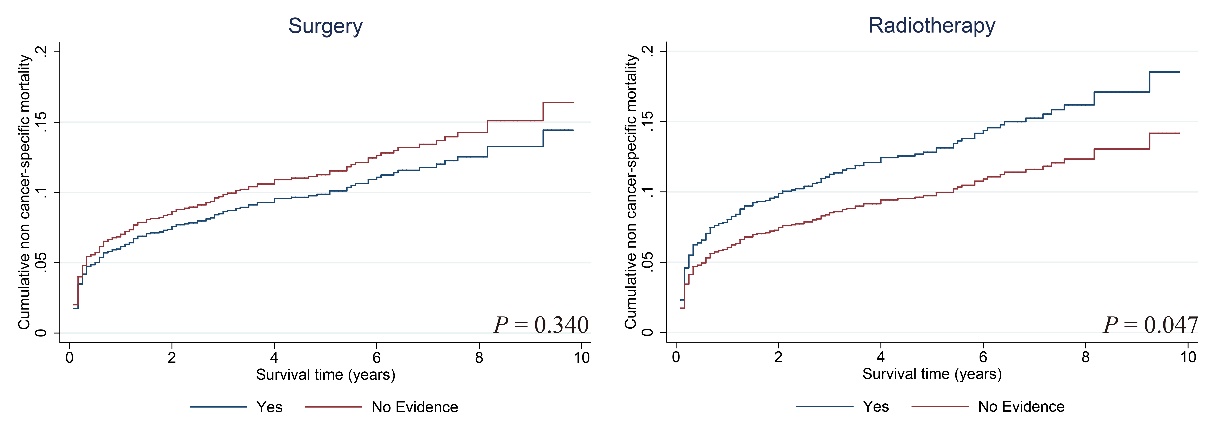


**Figure S4. Cumulative non-cancer-specific mortality in patients with primary central nervous system diffuse large B-cell lymphoma (negative results)**
